# How much should we still worry about QTc prolongation in rifampicin-resistant tuberculosis? ECG findings from TB-PRACTECAL clinical trial

**DOI:** 10.1128/aac.00536-24

**Published:** 2024-06-06

**Authors:** Ilaria Motta, Martina Cusinato, Andrew J. Ludman, Nathalie Lachenal, Matthew Dodd, Moe Soe, Tleubergen Abdrasuliev, Ruzilya Usmanova, Ilhomjon Butabekov, Tigay Zinaida Nikolaevna, Irina Liverko, Nargiza Parpieva, Ronelle Moodliar, Varvara Solodovnikova, Emil Kazounis, Bern-Thomas Nyang'wa, Katherine L. Fielding, Catherine Berry

**Affiliations:** 1 Médecins Sans Frontières, London, United Kingdom; 2 Department of Infectious Disease Epidemiology, London School of Hygiene & Tropical Medicine (LSHTM), London, United Kingdom; 3 Royal Devon University Healthcare NHS Foundation Trust, Exeter, United Kingdom; 4 Médecins Sans Frontières, Geneva, Switzerland; 5 Department of Medical Statistics, London School of Hygiene & Tropical Medicine (LSHTM), London, United Kingdom; 6 Médecins Sans Frontières, Amsterdam, the Netherlands; 7 Republican Phthisiological Hospital, Nukus, Karakalpakstan, Uzbekistan; 8 Republican Specialized Scientific and Practical Medical Center of Phthisiology and Pulmonology, Tashkent, Uzbekistan; 9 Republican Phthisiological Hospital 2, Nukus, Karakalpakstan, Uzbekistan; 10 THINK (TB&HIV Investigative Network): Doris Goodwin Hospital, Pietermaritzburg and Hillcrest, Durban, South Africa; 11 Republican Research and Practical Centre for Pulmonology and TB, Minsk, Belarus; St. George's, University of London, London, United Kingdom

**Keywords:** bedaquiline, BPaL/M, DR-TB, drug-resistant tuberculosis, cardiotoxicity

## Abstract

Regimens for the treatment of rifampicin-resistant tuberculosis currently rely on the use of QT-prolonging agents. Using data from the randomized controlled trial, TB-PRACTECAL, we investigated differences in QTcF among participants in the three interventional arms: BPaL (bedaquiline, pretomanid, and linezolid), BPaLC (BPaL with clofazimine), and BPaLM (BPaL with moxifloxacin). Additionally, we assessed whether age, body mass index, and country were causally associated with QTcF prolongation. The trial included participants from South Africa, Uzbekistan, and Belarus. A *post hoc* analysis of electrocardiogram data was undertaken. Random effects regression was used to model QTcF longitudinally over 24 weeks and causal frameworks guided the analysis of non-randomized independent variables. 328 participants were included in BPaL-based arms. The longitudinal analysis of investigational arms showed an initial QTcF steep increase in the first week. QTcF trajectories between weeks 2 and 24 differed slightly by regimen, with highest mean peak for BPaLC (QTcF 446.5 ms). Overall, there were 397 QTcF >450 ms (of 3,744) and only one QTcF >500 ms. The odds of QTcF >450 ms among participants in any investigational arm, was 8.33 times higher in Uzbekistan compared to Belarus (95% confidence interval: 3.25–21.33). No effect on QTcF prolongation was found for baseline age or body mass index (BMI). Clinically significant QTc prolongation was rare in this cohort of closely monitored participants. Across BPaL-based regimens, BPaLC showed a slightly longer and sustained effect on QTcF prolongation, but the differences (both in magnitude of change and trajectory over time) were clinically unimportant. The disparity in the risk of QTc prolongation across countries would be an important factor to further investigate when evaluating monitoring strategies.

**CLINICAL TRIALS:**

This study is registered with ClinicalTrials.gov as NCT02589782.

## INTRODUCTION

Rifampicin-resistant tuberculosis (RR-TB) is a significant global public health challenge with around 410,000 people falling ill annually (1). New and safer regimens were desperately needed to arrest this emerging epidemic.

QT interval is one of the most important markers of cardiac safety in drug development. QT interval corresponds to the electrical depolarisation and repolarisation of the ventricles captured on the electrocardiogram (ECG). The QT interval corrected by Fridericia’s formula (QTcF) is recommended. QTcF prolongation above 500 ms is associated with an increased risk for “torsade de pointes” (TdP), a polymorphic ventricular tachycardia that is a recognized cause of sudden death (2).

Well-known acquired and potentially modifiable factors that influence the QT interval are electrolyte imbalance, impaired renal or hepatic function, hypothyroidism, bradycardia, and concomitant use of QT-prolonging drugs. Congenital and non-modifiable factors that play a role in QT prolongation include sex (female), age, and congenital long QT syndrome (3). More recently, it has been suggested that a high baseline QT value is also a risk factor for drug-induced QT prolongation (4).

Cardiac safety has become an area of interest for TB physicians. Unfortunately, second-line anti-TB drugs are known to cause QT interval prolongation, including bedaquiline, clofazimine, fluoroquinolones, and delamanid. At least two, and often three such agents are used together in recommended regimens (5–7). In the initial C208 phase IIB study on the efficacy and safety of bedaquiline, the more frequent QT prolongation events and deaths in the bedaquiline arm brought this issue to the fore for RR-TB treatment (8). None of the deaths were considered cardiac or associated with high bedaquiline concentrations (8, 9).

The efficacy of 6-month regimens containing bedaquiline, pretomanid, and linezolid (BPaL) with and without moxifloxacin (BPaLM) has been established by phase III studies (10, 11). BPaLM is the current preferred option for RR-TB treatment without additional fluoroquinolone resistance according to the 2022 WHO guidelines (12).

In the TB-PRACTECAL clinical trial, one of the studies that gave evidence for new recommendations, intensive cardiac monitoring was undertaken to prevent and mitigate cardiac events and characterize the cardiac safety profile of the regimens. The trial found that both peak and week 24 QTcF were significantly higher in the standard of care (SOC) arm compared to the three investigational arms [BPaLM, BPaL-Clofazimine (BPaLC), and BPaL]. Mean differences from the SOC arm at week 24 were –18.1, –5.4, and −20 ms in BPaLM, BPaLC, and BPaL, respectively (10).

Further analysis is required to better understand the evolution of QT prolongation in the BPaL-based investigational regimens as well as identify the contribution of known risk factors in this cohort. Regional variation was also identified in the STREAM cohort, and we were keen to further investigate if this was a cofactor (13).

Therefore, we conducted analyses of TB-PRACTECAL ECGs to (i) evaluate the risk of QTcF prolongation across investigational regimens longitudinally over the treatment period and (ii) assess if country of enrolment, baseline age or BMI were risk factors for QTcF prolongation in investigational and standard of care arms.

## MATERIALS AND METHODS

### Study design, setting, and population

This is a longitudinal *post hoc* analysis of TB-PRACTECAL trial (NCT02589782) data to investigate the risk of QTcF prolongation and factors causally associated with QTcF measurements following treatment initiation.

TB-PRACTECAL was an open-label, phase 2–3, multicenter, randomized, controlled, non-inferiority trial to evaluate the efficacy and safety of three 24-week, all-oral regimens compared with the locally accepted SOC for the treatment of RR-TB. Participants were randomly assigned, in a 1:1:1:1 ratio, to BPaLM, BPaLC, BPaL, or SOC arm. The SOC was administered over 36–96 weeks. The trial was conducted in Belarus, South Africa, and Uzbekistan between January 2017 and March 2021. Exclusion criteria relevant for cardiac safety were: QTcF >450 ms, risk factors for QT prolongation or TdP, history of cardiac disease, syncopal episodes, significant symptomatic, or asymptomatic arrhythmias (except sinus arrhythmia) (10).

The population included in this analysis are participants aged ≥15 years with RR-TB randomized in TB-PRACTECAL before 1 September 2020, who received at least one dose of medication.

### Data sources, outcome, and covariates

At each study visit, pre-dose triplicate ECGs were recorded, and centrally reviewed by an electrophysiologist. Early in the trial pre- and post-dose ECGs were recorded. No important differences were observed between these time points, and only pre-dose ECGs were performed later on. Over the 108-week study period, 18 visits included ECGs as part of safety monitoring. Of these, 13 (at randomization, weekly between weeks 1 and 8, 4 weekly between weeks 12 and 24) took place during the first 24-week treatment period. For this analysis, pre-dose QTcF measurements at each scheduled visit were calculated as the mean of QTcF measurements, excluding those with recording errors. Unscheduled visits were excluded.

QTcF outcomes were analyzed using three measures: as a continuous outcome using repeated measures; a binary outcome [with >450 ms defining an event, equivalent to at least a grade 1 (mild) adverse event (14)] using repeated measures; and as the peak continuous value over the treatment period. All objectives evaluated the outcomes over the treatment period for the respective investigational arm. As the SOC had a variable duration, this arm was evaluated over 108 weeks.

Covariates of interest included demographics (sex, baseline age, and country), baseline QTcF, BMI, estimated glomerular filtration rate (eGFR) and potassium levels, hepatitis C and/or B virus (HCV/HBV) serology, and human immunodeficiency virus (HIV) status. In addition, other relevant comorbidities were considered; (i) arterial hypertension or cardiovascular disease (HTA/CVD) and (ii) diabetes mellitus (DM).

### Statistical methods and analysis

Two objectives were assessed: (i) the effect of the investigational regimens on QTcF prolongation over the treatment follow-up period (24 weeks); and (ii) the causal effect of country, age, and BMI on QTcF prolongation over 24 weeks for the investigational arms and over 108 weeks for the SOC arm. A full description of study objectives is available in (Appendix 1).

For objective 1, the QTcF prolongation continuous outcome was evaluated, as repeated measures, using linear regression, based on measurements from 0 to 24 weeks. The model included random effects on the intercept (baseline) and slopes (time variables) due to the repeated measures of the outcome. Interaction terms between the investigational arms and time variables were fitted, adjusting for country to account for the stratified randomization strategy of the main trial. Time was measured in weeks since randomization. Using fractional polynomials to model non-linear trends in time, we considered up to second-degree polynomial transformations (15). The best-fitting model identified had two non-linear time terms [1/time^2^ and ln(time)]. We also summarised QTcF levels as a binary variable, >450 ms, over the treatment follow-up period graphically.

Objective 2 evaluated (i) the direct effect of country, (ii) the total effect of age, and (iii) the total effect of low BMI, on QTcF prolongation. Age was categorised as 15–24, 25–34, 35–44, and ≥45 years and BMI as <18.5 and ≥18.5 kg/m^2^. Directed acyclic graphs (DAGs) were used to identify the sets of covariates for the adjustment of the independent variables, based on previous reports and *a priori* knowledge (3, 13, 16). Separate analyses were conducted for the combined investigational arms and the SOC, guided by different DAGs for these two groups (see Appendix A2). The categorization of treatments in the SOC arm was performed according to time of enrolment (pre- or post-implementation of the 2019 WHO recommendations to phase out injectables) and type of regimen (long or shorter) as described in Supplementary Appendix S5.3 in Nyang’wa et al. (10)

All continuous covariates were centerd at their mean. For each independent variable, the QTcF prolongation outcome was evaluated as (i) the risk of QTcF >450 ms over the treatment follow-up period, using a logistic model that accounted for repeated measures per participant through a random effect, and (ii) the peak QTcF value post-randomization, over the treatment follow-up period, (one outcome measure per participant), using a linear regression model.

This was a *post hoc* analysis with no *a priori* study size calculation. Missing data were not imputed because of a low level of missing data. Data management and statistical analyses were carried out using R (R Core Team, version 3.6.3, Vienna, Austria. Packages: tidyverse, lubridate, ggplot2, mfp).

## RESULTS

A total of 439 participants were recruited to the TB-PRACTECAL trial before 1 September 2020 and received at least one dose of medication. Of these, 328 were allocated to an investigational arm and 111 to the SOC. Two participants allocated to the SOC were excluded from this analysis: one with screening QTcF >450 ms and another with dextrocardia.

Baseline characteristics are reported in Table 1, by arm. In the investigational arms combined, 57.9% (190) were male, and the mean age was 35.7 years. Among these participants (328), there was a total of 3,744 ECGs over the 24-week treatment period; 0.3% (1 in BPaLC) reported QTcF >500 ms (grades 3–4 event), 3.4% (11) reported QTcF >480 ms (grade 2 event), and 31.1% (102) QTcF >450 ms (Table 2).

**TABLE 1:** Summary of baseline characteristics across study arms^*a*^

Variables		BPaLM *N* = 112	BPaLC *N* = 108	BPaL *N* = 108	SOC *N* = 109
Country	Belarus	20 (17.9%)	19 (17.6%)	20 (18.5%)	19 (17.4%)
	South Africa	39 (34.8%)	38 (35.2%)	36 (33.3%)	38 (34.9%)
	Uzbekistan	53 (47.3%)	51 (47.2%)	52 (48.1%)	52 (47.7%)
Sex at birth	Male	63 (56.2%)	71 (65.7%)	56 (51.9%)	65 (59.6%)
	Female	49 (43.8%)	37 (34.3%)	52 (48.1%)	44 (40.4%)
Age (years)	Mean (SD)	36.1 (11.6)	33.5 (10.7)	37.5 (11.6)	38.4 (10.9)
	Range	18.2–61.2	15.5–68.0	15.7–72.5	19.1–71.2
BMI (kg/m^2^)	Mean (SD)	20.8 (4.7)	20.2 (4.2)	20.8 (3.7)	20.4 (3.9)
	Range	15.6–47.2	13.2–37.6	13.4–33.0	14.6–37.9
HIV status	Negative	83 (74.1%)	85 (78.7%)	74 (68.5%)	81 (74.3%)
	Positive	29 (25.9%)	23 (21.3%)	34 (31.5%)	28 (25.7%)
HBV/HCV	Negative	104 (92.9%)	97 (89.8%)	93 (86.1%)	95 (88.8%)
	Positive	8 (7.1%)	11 (10.2%)	15 (13.9%)	12 (11.2%)
	Missing	0	0	0	2
eGFR (mL/min)	Mean (SD)	112.1 (30.4)	117.4 (37.1)	111.2 (35.0)	112.2 (41.2)
	Range	43.2–216.0	61.4–340.3	48.6–224.5	44.4–312.5
	Missing	0	1	0	1
Potassium (mmol/L)	Mean (SD)	4.4 (0.5)	4.4 (0.4)	4.4 (0.5)	4.3 (0.4)
	Range	3.4–5.7	3.5–5.8	3.3–5.8	3.4–5.3
	Missing	0	1	0	0
DM	Negative	110 (98.2%)	104 (96.3%)	103 (95.4%)	104 (95.4%)
	Positive	2 (1.8%)	4 (3.7%)	5 (4.6%)	5 (4.6%)
HTA/CV	Negative	106 (94.6%)	98 (90.7%)	99 (91.7%)	101 (92.7%)
	Positive	6 (5.4%)	10 (9.3%)	9 (8.3%)	8 (7.3%)

^*a*^ SOC: standard of care. SD: standard deviation. BMI: body mass index. HTA/CV: hypertension/cardiovascular disease. DM: diabetes mellitus. eGFR: estimated glomerular filtration rate.

**TABLE 2:** Summary of QTcF measures at baseline, and over 24 weeks for the investigational arms and 108 weeks for the standard of care (SOC)^*d*^

Variables		BPaLM *N* = 112	BPaLC *N* = 108	BPaL *N* = 108	SOC^*a*^ *N* = 109
QTcF measurements (#visits)	Mean (SD)	11.5 (2.4)	11.5 (2.5)	11.3 (2.5)	12.9 (4.5)
	Range	3–13	2- 13	1–13	2–18
QTcF baseline (ms)	Mean (SD)	396.2 (17.7)	395.3 (19.7)	398.4 (18.5)	400.4 (18.6)
	Range	354.3–437.0	356.3–442.7	351.0–436.0	343.0–455.3
	Missing	3	0	1	0
QTcF peak in follow-up (ms)	Mean (SD)	439.5 (20.7)	446.5 (19.4)	436.0 (22.2)	457.5 (21.8)
	Range	394.7–488.3	409.3–502.7	367.7–488.7	408.0–518.7
QTcF mean^*b*^ (ms)	Mean (SD)	420.7 (17.8)	424.0 (17.1)	418.8 (18.7)	430.4 (17.8)
	Range	382.7–460.6	380.7–460.0	367.7–469.9	386.2–474.9
Participants^*c*^	No	79 (70.5%)	66 (61.1%)	81 (75.0%)	38 (34.9%)
QTcF >450 ms	Yes	33 (29.5%)	42 (38.9%)	27 (25.0%)	71 (65.1%)
Participants^*c*^	No	108 (96.4%)	104 (96.3%)	105 (97.2%)	94 (86.2%)
QTcF >480 ms	Yes	4 (3.6%)	4 (3.7%)	3 (2.8%)	15 (13.8%)
Participants^*c*^	No	112 (100.0%)	107 (99.1%)	108 (100.0%)	106 (97.2%)
QTcF >500 ms	Yes	0 (0.0%)	1 (0.9%)	0 (0.0%)	3 (2.8%)
Events (ECGs)	No	1,165 (90.7%)	1,054 (85.2%)	1,128 (92.3%)	1,095 (77.6%)
>450 ms	Yes	120 (9.3%)	183 (14.8%)	94 (7.7%)	316 (22.4%)
	Total	1,285 (100.0%)	1,237 (100.0%)	1,222 (100.0%)	1,411 (100.0%)

^*a*^ Variables for the standard of care (SOC) group summarised over 108 weeks and the variables for the interventional regimen over 24 weeks. The values for the interventional regimens refer to objectives 1 and 2. The values for SOC refer to the additional analysis in this population (objective 2).

^*b*^ Mean QTcF for each patient over all their follow-up visits, and then a mean for that for each arm.

^*c*^ Participants with at least one event. Number of events refers to the total number of QTcF evaluated. ms: milliseconds.

^*d*^ The table summarises QTcF at an individual/participant level. SD: standard deviation.

In the SOC arm (109), participants contributed 1,411 ECGs over the 108-week follow-up period; 65 (59.6%) were male and mean age was 38.4 years. Among participants enrolled to the SOC (109), 2.8% (3), 13.8% (15), and 65.1% (71) reported QTcF >500 ms, QTcF >480 ms, and QTcF >450 ms, respectively.

### Objective 1: What is the risk of QTcF prolongation during treatment for BPaLM, BPaLC, and BPaL?

The number of QTcF measures over 24 weeks was similar across the investigational arms [mean 11.4, standard deviation (SD) 2.5, median 13, interquartile range 10 to 13] as were the QTcF values at baseline (mean 396.6, SD 18.6 ms). The peak QTcF value observed over 24 weeks was highest for BPaLC (mean 446.5, SD 19.4 ms), followed by BPaLM (mean 439.7, SD 20.7 ms) and then BPaL (mean 436.0, SD 22.2 ms), Table 2 and Appendix 3.

**Fig 1:**
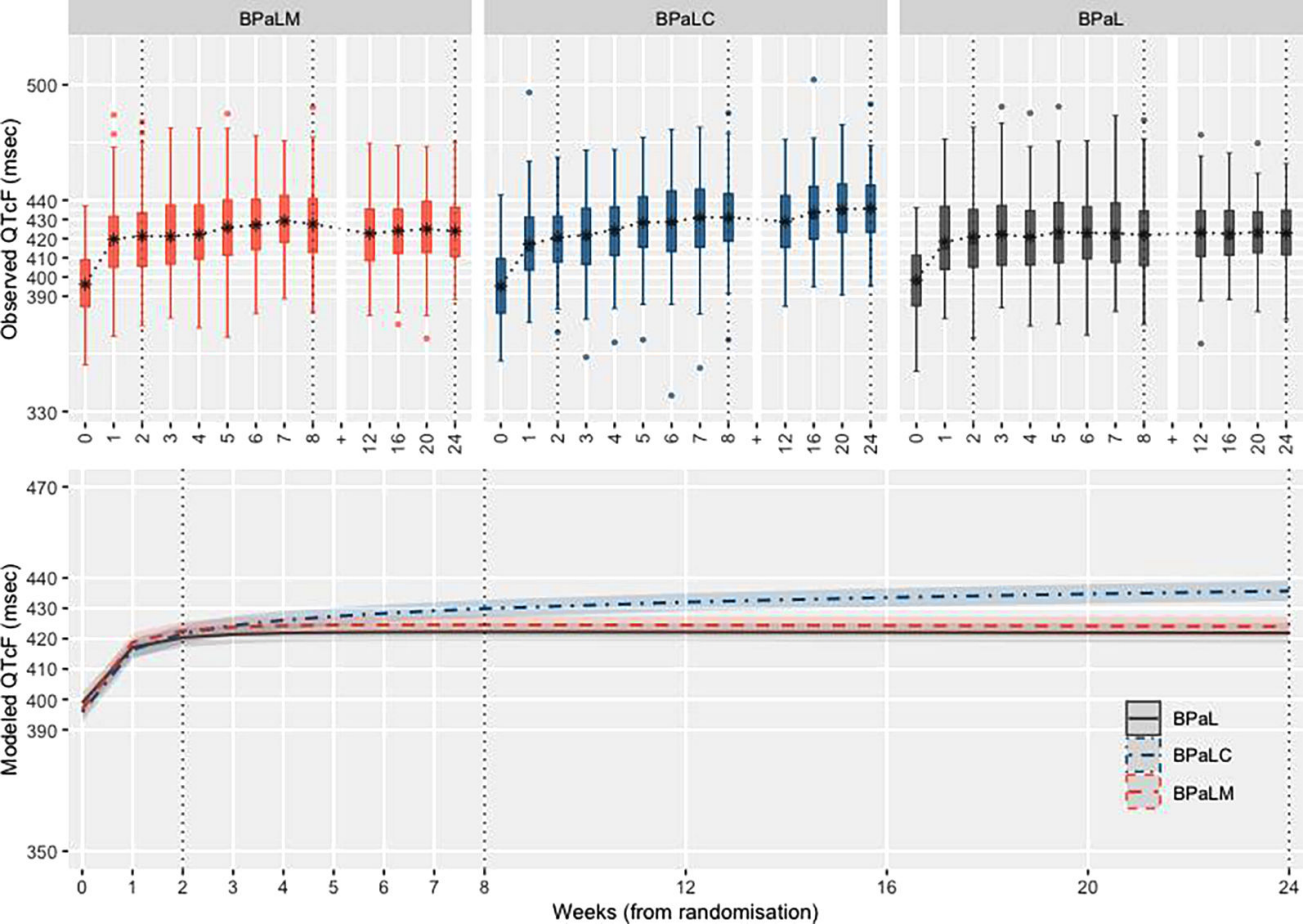
Observed and modeled QTcF values over the 24-week follow-up time. Top panel: Observed QTcF summarised as box plots. Time points from 0 to 24 weeks are equally spaced and do not reflect the true spacing between visits and the *y*-axis extends from 330 to 550 ms. The asterisks indicate the mean. The symbol ‘+’ on the *x*-axis represents a change of scale from 1 to 4 weeks. These data, plotted as spaghetti plots for *x*-axis as continuous scale, from 0 to 108 weeks, are included in (Appendix A5). Bottom panel: Modeled QTcF values, with the *x*-axis extending from 0 to 24 weeks (with time points reflecting the true spacing between visits) and the *y*-axis extending from 350 to 470 ms. Strong evidence for interaction between BPaLC and the second term for time [ln(time)] (*P* value < 0.001), is indicated by non-parallel trajectories of QTcF for BPaLC after the turning point.

The observed distribution of QTcF values per visit/week is shown in Fig. 1 (top panel). QTcF values increased sharply in the first week for all investigational arms. Between weeks 2 and 24, QTcF trajectories differed by regimen. After week 24, following the end of treatment, observed QTcF values showed a downward trajectory for all regimens (Appendix 5; Fig. S1, S2, and S3).

Modeled QTcF values are shown in the bottom panel in Fig. 1. In the BPaLC arm, QTcF values progressively increased over the 24 weeks, with modeled means at 8, 12 and 24 weeks of 429.9, 432.0 and 435.7 ms, respectively. In the BPaLM and BPaL arms, QTcF values were constant between 6 and 24 weeks, though marginally higher in the BPaLM versus the BPaL arm (BPaLM: modeled means at 8, 12, and 24 weeks of 424.6, 424.5, and 424.0 ms, respectively; BPaL modeled means at 8, 12, and 24 weeks of 422.2, 422.2, and 421.9 ms, respectively). See the supplemental materials (Appendix 6; table S1 and Fig. S4) for model output and diagnostics.

For QTcF prolongation as a binary outcome, the percentage of participants who experienced at least one event of QTcF >450 ms over 24 weeks of treatment was 29.5% (33/112), 38.9% (42/108), and 25.0% (27/108) in the BPaLM, BPaLC, and BPaL arms, respectively ( Table 2).

Out of 120 QTcF >450 ms events registered in BPaLM, 85.8% (103) occurred by week 12. This proportion was 68.3% (125/183) and 80.8% (76/94) for BPaLC and BPaL, respectively (Appendix 4 and Appendix 5; Fig. S4).

### Objective 2: Is there an effect of country of enrolment, age, and BMI on QTcF prolongation?

The results for the outcome considered as a binary repeated event (QTcF >450 ms) and as peak QTcF, for the investigational arms, are shown in Table 3. Uzbekistan reported a larger absolute (and relative) non-baseline number of events (315 QTcF >450 ms out of 1,669 ECGs recorded, 18.9%) compared to Belarus (48 events from 638 ECGs, 7.5%) and South Africa (25 events from 1,058 ECGs, 2.4%). The odds of QTcF >450 ms in each participant in the investigational arms, adjusting for regimen, baseline QTcF, and eGFR, were 8.33 [95% confidence interval (CI): 3.25–21.33] times higher in Uzbekistan compared to Belarus, and similar among South African and Belarus participants [odds ratio (OR): 0.80, 95% CI: 0.26–2.50]. Consistent results were observed when analyzing peak QTcF value (Table 3). Peak QTcF values in Uzbekistan were 11.91 ms higher than in Belarus (95% CI: 7.40–16.43), adjusting for regimen, baseline QTcF, and eGFR.

**TABLE 3:** Model results exploring the direct effect of country and the total effect of age and BMI in the investigational arms (over 24 weeks)^*a*^

Model	*N^b^*_e_	QTcF^*c*^	aOR^*d*^	95% CI	Np^*f*^	Mean^*g*^	aQTcF-max^*h*^	95% CI
Country^*i*^								
Belarus	638	48	1.00	–^*e*^	59	439.2	0.00	–
South Africa	1,058	25	0.80	(0.26 to 2.50)	110	429.1	−2.79	(−7.69 to 2.11)
Uzbekistan	1,669	315	8.33	(3.25 to 21.33)	154	449.3	11.91	(7.40 to 16.43)
Age (years)^*j*^								
15–24	591	57	0.70	(0.16 to 3.06)	56	439.8	−2.09	(−8.88 to 4.71)
25–34	1,192	145	1.00	–	115	441.9	0.00	–
35–44	908	108	0.64	(0.18 to 2.32)	90	438.0	−3.87	(−9.74 to 1.99)
≥45	729	87	2.24	(0.51 to 9.75)	67	442.7	0.75	(−5.66 to 7.15)
BMI (kg/m^2^)^*k*^								
≥18.5	2,260	242	1.00	–	218	439.0	0.00	–
<18.5	1,160	155	1.44	(0.59 to 3.52)	110	444.0	4.27	(−0.15 to 8.68)

^*a*^ There were four participants with missing values of baseline QTcF and one with missing values of baseline eGFR. There was one participant (with no missing values on baseline variables) who was discontinued immediately after randomisation (baseline) due to not meeting inclusion/exclusion criteria. As this participant only has baseline outcome data, they are excluded from the random effect models. The mixed models include one person fewer than the linear model.

^*b*^ Ne: Total number of events/ECGs.

^*c*^ QTcF >450ms.

^*d*^ aOR: adjusted OR estimated using logistic regression, excluding baseline outcome.

^*e*^ –: baseline.

^*f*^ Np: total number of participants.

^*g*^ Mean of peak QTcF (ms).

^*h*^ aQTcF-max: adjusted difference in peak QTcF value in ms, estimated using linear regression.

^*i*^ Models estimating the direct effect of country, adjusting for baseline QTcF (ms), baseline eGFR, regimen (Ne = 3365, Np = 323).

^*j*^ Models estimating the total effect of age, no adjustment needed. (Ne = 3420, Np = 328). Modal category has been used as baseline to increase the precision of the estimates.

^*k*^ Models estimating the total effect of low BMI (<18.5 kg/m2) adjusting for country and sex (Ne = 3420, Np = 328).

There was no evidence that baseline age or BMI <18.5 kg/m^2^ influenced QTcF >450 ms, nor that age was associated with the peak QTcF. There was, however, a suggestion that BMI <18.5 kg/m^2^ at baseline was associated with higher peak QTcF [difference of 4.27 ms (95% CI: −0.15 to 8.68) vs normal BMI] after controlling for the effect of country and sex, though the 95% CI included 0.

For the SOC population, the models showed consistent results for the effect of age and BMI on both QTcF >450 ms and peak QTcF; in contrast, there was no association between the country of enrolment and QTcF modeled as QTcF >450 ms and peak QTcF, after adjusting for the SOC regimen, baseline QTcF, and eGFR. However, the point estimates were in the same direction as in the investigational arms country effect analysis (Appendix 8).

## DISCUSSION

In this *post hoc* analysis of ECGs from TB-PRACTECAL participants, we investigated the risk of QTcF interval prolongation across BPaLM, BPaLC, and BPaL and the effect of country of enrolment, age, and BMI on QTcF—separately, in the interventional and the SOC arms. We observed few QT-prolonging events with BPaL-based regimens, and for scheduled monitoring, only one instance of QTcF greater than 500 ms, in a participant on BPaLC.

The analysis of our first objective showed that during the treatment period (24 weeks), the peak QTcF was highest for those on BPaLC, followed by BPaLM and then BPaL. The longitudinal analysis observed an initial steep increase of QTcF in the first week, followed by smaller increments over the second week for all investigational arms. These data add to the previous analyses of cardiac safety in bedaquiline-containing regimens.

In a prospective South African cohort (PROBeX study), Brust et al., investigated QT in patients receiving longer regimens, most of whom received bedaquiline, clofazimine, and fluoroquinolone. ECG monitoring in the study was performed at baseline, and at months 1, 2, and 6. In their cohort, the mean QTcF increased from 404.6 ms at baseline to 427.6 ms at month 6, with 23% of participants recording at least one event QTcF >450 ms (17). Despite the regimens in PROBeX study resembling the SOC used in TB-PRACTECAL, the effect on QTcF compares more closely to TB-PRACTECAL investigational arms where the mean QTcF at 6 months were 423.9, 435.6, and 423.0 ms in BPaLM, BPaLC, and BPaL, respectively (Appendix 3). In the TB-PRACTECAL cohort, 65.1% of SOC participants had a recorded QTcF >450 ms (vs 23% in Brust cohort). Monitoring was far more frequent in TB-PRACTECAL.

With respect to the higher peak QTcF observed for BPaLC (compared to BPaLM and BPaL), these findings were consistent with those of Pym et al. In their analysis, addition of clofazimine to a bedaquiline-containing optimized background regimen showed a greater increase (31.9 ms) of QTcF interval from baseline to week 24, compared to no addition (12.3 ms) (18). Also, similar to Brust’s cohort, in the TB-PRACTECAL BPaLC arm, QTcF values continued to rise over the 24 weeks, although slightly after 8 weeks. Isralls et al., in a retrospective cohort study of programmatic use of bedaquiline in South Africa, found that the greatest QTcF increase was in the first 4–6 weeks and the highest odds of QTcF >500 ms (or QTcF >60 ms change from baseline) was at week 15 (19).

Interestingly, van Beek et al. using PROBeX data, simulated a strategy of ECG monitoring and found that the vast majority of patients that should have interrupted bedaquiline treatment due to a QTcF interval >500 ms had already been identified by week 12 (20).

Similarly, in our study, the majority of QTcF events >450 ms occurred for participants receiving BPaLM (85.8%) and BPaL (80.8%) by week 12. For participants receiving BPaLC, the proportion of QTcF events >450 ms by week 12 was lower (68.3%), potentially suggesting in case of concomitant treatment with bedaquiline and clofazimine longer ECG monitor during the treatment.

As part of our second objective, we investigated the direct effect of country (i.e., not mediated by other factors) on the risk of QTcF prolongation. Participants enrolled in investigational arms in Uzbekistan were found to have higher risk of QTcF >450 ms (OR: 8.33, 95% CI: 3.25–21.33) and higher peak QTcF over 24 weeks (adjusted mean difference 11.91 ms, 95% CI: 7.40–16.43) compared to Belarus after accounting for baseline QTcF, baseline eGFR, and TB regimen. Given the observed homogeneity of race within the country (in Belarus 100% were White, in South Africa 98% were Black, and in Uzbekistan 95% were Asian), we considered the effect of country explained, at least partially, by this variable.

In their analysis, Brust et al. found black race a potential predictor of QTcF >450 ms [aOR: 3.2 (95% CI: 0.97–10.41)] (17). A regional variation was reported in STREAM Stage 1 trial with a higher proportion of events of QTcF >500 ms in participants enrolled in Mongolia (45.5% compared to 3.5 and 11.9% from other sites), and who also had higher baseline QT (13). In STREAM Stage 1, the investigational arm included clofazimine and a fluoroquinolone, but not bedaquiline.

Our finding aligns with results from STREAM Stage 1 and could be due to site-related environmental factors, regional genetic predisposition, or presence of single nucleotide polymorphisms variation for genes encoding for drug transporters affecting the pharmacokinetics of QT-prolonging drugs (21).

In our study, the country effect was not found to be statistically significant in the SOC population despite containing similar drugs (bedaquiline, fluoroquinolone, and clofazimine), although the direction of the point estimates was consistent with that of the interventional arms. Reasons for the inconsistency could be related to the multiplicity of the SOC regimens used at different sites, diluting the country effect; the SOC regimens differed across sites and changed over time due to changes in WHO recommendations. For example, only 26.9% (14/52) of participants enrolled in the SOC in Uzbekistan received shorter regimens (9–11 month regimens with and without an injectable agent), compared to 68.4% (26/38) in South Africa and none in Belarus (Appendix 7). Other reasons could be the coarse categorizations used for the SOC (length and time of enrolment, before or after 2019 WHO guidelines) (10), the smaller sample for the SOC arm compared to the total of participants in investigational arms (109 vs 328 subjects, respectively) and residual confounding. Further analyses are required to confirm our findings.

Also, as part of the second objective, we found no clear evidence of an effect of age on QTcF (>450 ms and maximum QTcF) which is inconsistent with reports of increased QTcF in older patients (3, 16, 17). This could be explained by the relatively young population included in our study (only 21.7% were above 45 years old). Low baseline BMI was found to be a risk factor for slightly higher peak QTcF values but not for QTcF events >450 ms. Participants in the investigational arm with low BMI had, on average, a maximum QTcF 4.27 ms higher than participants with normal/high BMI (95% CI: −0.15 to 8.68 ms), though the confidence interval included 0. In the SOC arm, the magnitude of this effect was larger (average peak QTcF 9.03 ms, 95% CI: 0.62–17.44). This may support an individual-based approach to ECG monitoring, with increased frequency reserved for low BMI patients, even if the magnitude of increase is minimal and probably not clinically meaningful. Jayanti et al. found that QTc was increased among overweight and obese groups compared to normal BMI, hypothesizing an altered autonomic homeostasis (22). It would be interesting to further investigate the association we found between QTcF and low BMI and whether or not it is mediated by higher TB drug-exposure in this subgroup. Separate PK analyses are ongoing and potentially could explain this finding (23).

To our knowledge, this is the first study reporting QTcF prolongation in BPaL-based regimens from a large clinical trial where all participants, irrespective of QTcF measurements, were intensively monitored through ECGs (at each visit) that were centrally reviewed by electrophysiologists. For the present analysis, we used DAGs to represent qualitative expert knowledge and *a priori* assumptions about the causal structures underlying our research questions. DAGs are the well-known strategy to identify potential sources of bias and structure of confounding (24).

Our population is more geographically diverse than much of the published literature, but we acknowledge that participants were at lower risk of QT interval prolongation based on the clinical trial exclusion criteria (i.e., QTcF >450 ms at baseline, risk for QT prolongation, and history of cardiac disease). Consequently, we observed few severe QTcF prolongations (>500 ms), and the binary outcome threshold was set at 450 ms (grade 1 event).

The absence of a parameter for concomitant medications is a limitation in the analysis, but the study protocol restricted the administration of known QT-prolonging agents such as selected antipsychotics, macrolides, or prolonged use of azoles. Residual confounding introduced by contextual assumptions made for DAGs cannot be ruled out. In our cohort, per internal guidelines, participants who experienced grade 2 events (QTcF >480 ms) had more frequent unscheduled ECG monitoring. To avoid bias, unscheduled ECGs were excluded from the analyses, but we recognize in clinical practice an abnormal ECG would trigger a more intensive monitoring strategy.

Future research priorities should include a better understanding of geographical differences in QT prolongation risk with drug exposure data and an evaluation of the optimal ECG monitoring strategy. A strategy that would allow more targeted monitoring to decrease the number of ECGs needed during treatment would be beneficial, especially in resource-limited settings.

In conclusion, BPaL-based regimens caused few and predominantly mild QT-prolongation events in this selected, clinical trial cohort.

Participants receiving BPaLC experienced higher peak QTcF values compared to those receiving BPaL and BPaLM regimens. The small differences in mean QTcF (per visit) and trajectories across the three arms have limited clinical importance in our setting and were not associated with adverse events.

Intensive ECG monitoring is not warranted for most patients and the safe cardiac profile of BPaL-based regimens should reassure clinicians as they transition patients to new recommended regimens.

## SUPPLEMENTAL MATERIAL

Supplemental Appendix A3 aac.00536-24-s0001.xlsx Summary of observed QTcF and modeled QTcF.

Supplemental material aac.00536-24-s0002.docx Appendices A1, A2, A4 to A8; Fig. S1 to S5.

ASM does not own the copyrights to Supplemental Material that may be linked to, or accessed through, an article. The authors have granted ASM a non-exclusive, world-wide license to publish the Supplemental Material files. Please contact the corresponding author directly for reuse.
