# Supplementary material for: How much should we still worry about QTc prolongation in rifampicin-resistant tuberculosis? ECG findings from TB-PRACTECAL clinical trial
Source: Antimicrob Agents Chemother. 2024 Jun 6;68(7):e00536-24. doi: 10.1128/aac.00536-24 (PMC11232376; doi:10.1128/aac.00536-24)

# APPENDICES

Appendices are shown in supplemental material.

A1 Full description of study objectives

A2 DAGs and model specifications for investigational and SOC arms

A3 Summary of observed QTcF, modelled QTcF (in Excel)

A4 Observed proportions of QTcF >450ms per visit/week

A5 Additional plots

A6 Longitudinal model for QTcF, output and diagnostics

A7 Standard of Care Details

A8 Model results for objective 2 in SOC arm (country, age, and BMI)

# A1 Full description of study objectives

The objectives of this study are:

1. To evaluate if investigational regimens including moxifloxacin (Mfx) or clofazimine (Cfz), BPaLM and BPaLC, increased the risk of raised QTcF values and QTcF >450ms over 24 weeks of follow up compared to BPaL regimen, among TB patients participating in TB-PRACTECAL.
   1. To evaluate if country of enrolment was an independent risk factor for QTcF >450ms, in adult TB patients (on treatment) participating to TB-PRACTECAL.
   2. To evaluate if age at baseline was risk factor for QTcF >450ms, irrespective of regimen, in adult TB patients (on treatment) participating to TB-PRACTECAL.
   3. To evaluate if body mass index (BMI) at baseline was risk factor for QTcF >450ms, irrespective of regimen, in adult TB patients (on treatment) participating to TB-PRACTECAL.

# A2 DAGs and model specifications for investigational arms and SOC

DAGs (directed acyclic graphs) encode causal assumptions (not questions to be answered). The assumptions of causality are denoted by arrows (a.k.a. directed edges); for example, A-> B means that A causally affects B in some way (although the relationship can follow any functional form).

Country, QT at baseline, potassium levels, gender, BMI, age, HIV and HCV/HBV status, presence of comorbidities (hypertension/cardiovascular diseases and diabetes) and eGFR were considered as covariates and for each of those ancestor(s) (direct or indirect cause) and descendant(s) (direct or indirect effect) variables were identified. The assumptions of causality were different for investigational and SOC arms due to the differential nature of the association between regimen and country, i.e. randomisation was stratified by country, but the standard of care was an heterogeneous intervention and varied across countries (in addition, HIV status could determine the choice of regimen within the standard of care). For this reason, separate DAGs (and models) were specified for these populations. The parsimony principle was used when more than one set of covariates were possible.

Here we show:

Part 1 shows a table including a breakdown of causal assumptions for each variable.

Part 2 shows two DAGs: one for the investigational arms and one for the SOC arm.

Part 3: shows model equations.

Part 1: table including a breakdown of causal assumptions for each variable

| **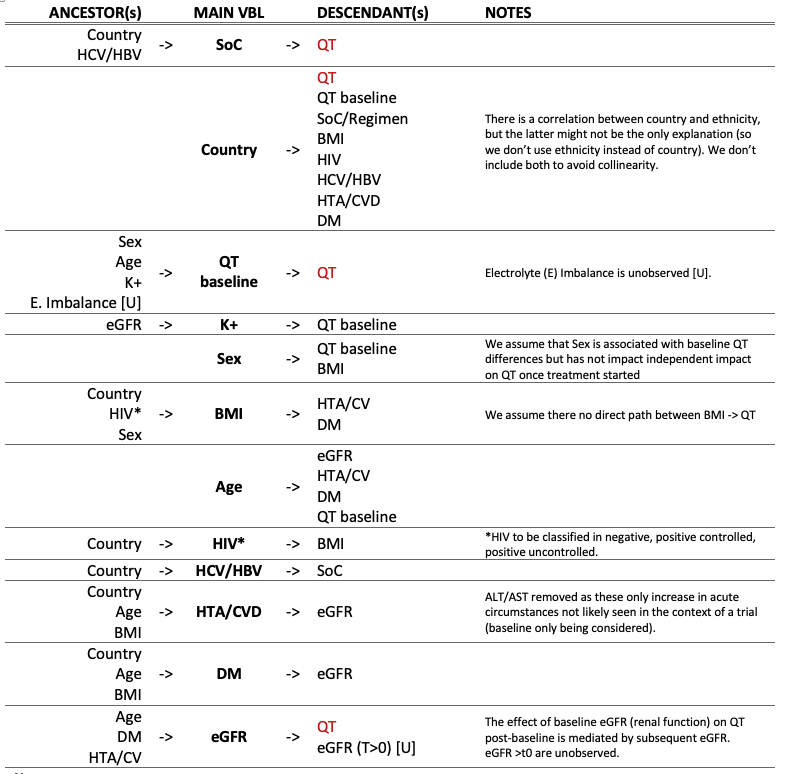** |
| --- |
| **Abbreviations:**  HCV: Hepatitis C virus. HBV: Hepatitis B virus. SoC: Standard of care. QT: QT corrected with Fridericia’s formula. BMI: Body mass index. HIV: human immunodeficiency virus. HTA: Hypertension. CV: Cardiovascular disease. DM: Diabetes mellitus. eGFR: estimated glomerular filtration rate. K+: Potassium. T>0: after baseline  **Notes**:  - [U]: unobserved variable (‘Electrolyte imbalance at time 0’) and ‘eGFR >t0 (after treatment start)’  - eGFR: estimated glomerular filtration rate estimates renal function. Estimated using Cockcroft-Gault CrCl, mL/min = (140 – age) × (weight, kg) × (0.85 if female) / (72 × Cr, mg/dL).  - It has been hypothesised that antihypertensive and antidiabetic treatment is associated with QT. However, at this stage this is a question and not an assumption that we can make. So, this is not included in our assumptions.  - Concomitant medications at baseline and QT at baseline have not been included. They might be clinically relevant but in this population I/E criteria ‘restrict’ the sample to individuals who are similar to each other with respect to those variables.  - Paths between (i) HIV -> HCV/HBV and (ii) Age -> BMI are not considered strong (biological) assumptions |

Part 2: DAGs (investigational and SOC arms)

| **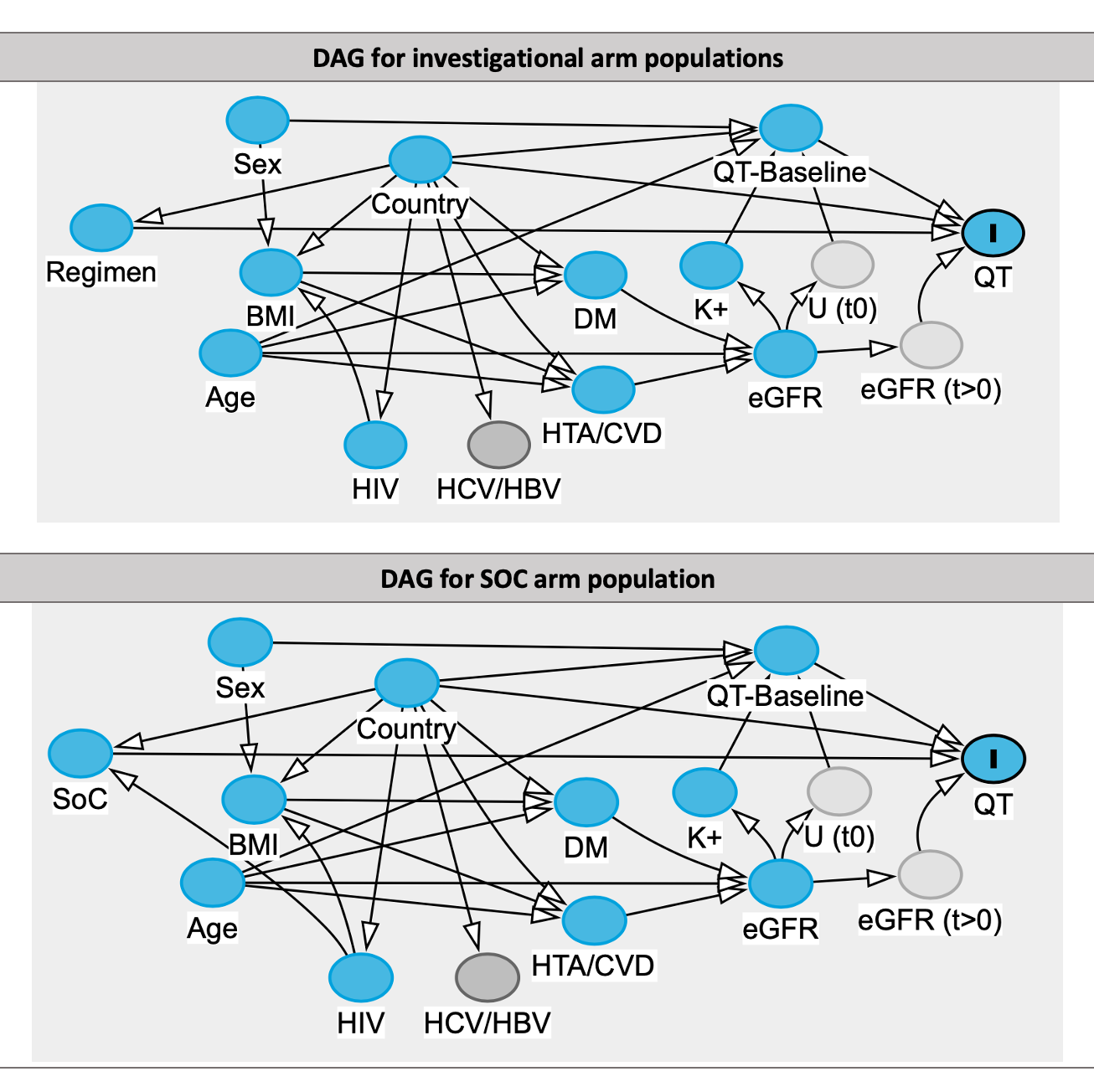** |
| --- |
| **Abbreviations:**  HCV: Hepatitis C virus. HBV: Hepatitis B virus. SoC: Standard of care. QT: QT corrected with Fridericia’s formula. BMI: Body mass index. HIV: human immunodeficiency virus. HTA: Hypertension. CV: Cardiovascular disease. DM: Diabetes mellitus. eGFR: estimated glomerular filtration rate. K+: Potassium. T>0: after baseline |

Part 3: Model specification

|  | **INTERVENTION** | **SOC** |
| --- | --- | --- |
| **OBJECTIVE 1** | Direct effect of Regimen on QT  Equation:  Outcome ~ time^-2 *regimen + ln(time) * regimen + country + RE | -- |
|  | | |
|  | **INTERVENTION** | **SOC** |
| **OBJECTIVE 2** | Direct effect of Country on QT  Equations:  Outcome ~ country + QT_baseline + eGFR_baseline + regimen | Direct effect of Country on QT  Equations:  Outcome ~ country + QT_baseline + eGFR_baseline + SOC |
|  | Total effect of Age on QT  Equations:  Outcome ~ age | Total effect of Age on QT  Equations:  Outcome ~ age |
|  | Total effect of BMI on QT  Equations:  Outcome ~ BMI + country + sex | Total effect of BMI on QT  Equations:  Outcome ~ BMI + country + sex + SOC |
| Abbreviations:  QT: QT corrected with Fridericia’s formula. QT450: QT over 450ms. QTmax: maximum QT value. ln: natural logarithm. RE: random effects. SOC: standard of care length/type.  Notes:  -For objective 2, the effect of baseline QTcF was assessed explicitly, so it was included as a fixed effect, which necessitated the exclusion of baseline visit from the outcome evaluated as repeated measures. | | |

# A3 summary of observed qtcf, modelled qtcf

See Excel

# A4 observed proportions of qtcf >450ms per visit/week

|  |  | BY | | | SA | | | UZ | | | OVERALL | | |
| --- | --- | --- | --- | --- | --- | --- | --- | --- | --- | --- | --- | --- | --- |
| Arm | Visit/W | QTcF  >450 | N | % | QTcF  >450 | N | % | QTcF  >450 | N | % | QTcF  >450 | N | % |
| BPaLM | 0 | 0 | 20 | 0.0 | 0 | 38 | 0.0 | 0 | 51 | 0.0 | 0 | 109 | 0.0 |
| BPaLM | 1 | 0 | 19 | 0.0 | 0 | 39 | 0.0 | 9 | 53 | 17.0 | 9 | 111 | 8.1 |
| BPaLM | 2 | 0 | 20 | 0.0 | 0 | 38 | 0.0 | 12 | 53 | 22.6 | 12 | 111 | 10.8 |
| BPaLM | 3 | 0 | 18 | 0.0 | 0 | 32 | 0.0 | 10 | 49 | 20.4 | 10 | 99 | 10.1 |
| BPaLM | 4 | 1 | 20 | 5.0 | 0 | 37 | 0.0 | 9 | 52 | 17.3 | 10 | 109 | 9.2 |
| BPaLM | 5 | 2 | 18 | 11.1 | 0 | 30 | 0.0 | 9 | 49 | 18.4 | 11 | 97 | 11.3 |
| BPaLM | 6 | 2 | 18 | 11.1 | 1 | 27 | 3.7 | 10 | 48 | 20.8 | 13 | 93 | 14.0 |
| BPaLM | 7 | 3 | 18 | 16.7 | 0 | 22 | 0.0 | 12 | 43 | 27.9 | 15 | 83 | 18.1 |
| BPaLM | 8 | 1 | 20 | 5.0 | 1 | 29 | 3.4 | 13 | 49 | 26.5 | 15 | 98 | 15.3 |
| BPaLM | 12 | 2 | 20 | 10.0 | 0 | 32 | 0.0 | 6 | 46 | 13.0 | 8 | 98 | 8.2 |
| BPaLM | 16 | 1 | 19 | 5.3 | 0 | 28 | 0.0 | 2 | 45 | 4.4 | 3 | 92 | 3.3 |
| BPaLM | 20 | 1 | 19 | 5.3 | 2 | 29 | 6.9 | 6 | 46 | 13.0 | 9 | 94 | 9.6 |
| BPaLM | 24 | 1 | 19 | 5.3 | 0 | 25 | 0.0 | 4 | 47 | 8.5 | 5 | 91 | 5.5 |
| BPaLC | 0 | 0 | 19 | 0.0 | 0 | 38 | 0.0 | 0 | 51 | 0.0 | 0 | 108 | 0.0 |
| BPaLC | 1 | 1 | 19 | 5.3 | 1 | 37 | 2.7 | 7 | 51 | 13.7 | 9 | 107 | 8.4 |
| BPaLC | 2 | 1 | 19 | 5.3 | 0 | 37 | 0.0 | 10 | 51 | 19.6 | 11 | 107 | 10.3 |
| BPaLC | 3 | 0 | 18 | 0.0 | 1 | 28 | 3.6 | 10 | 47 | 21.3 | 11 | 93 | 11.8 |
| BPaLC | 4 | 2 | 18 | 11.1 | 0 | 36 | 0.0 | 16 | 50 | 32.0 | 18 | 104 | 17.3 |
| BPaLC | 5 | 1 | 17 | 5.9 | 1 | 30 | 3.3 | 12 | 45 | 26.7 | 14 | 92 | 15.2 |
| BPaLC | 6 | 2 | 16 | 12.5 | 1 | 30 | 3.3 | 14 | 44 | 31.8 | 17 | 90 | 18.9 |
| BPaLC | 7 | 1 | 16 | 6.3 | 1 | 28 | 3.6 | 16 | 43 | 37.2 | 18 | 87 | 20.7 |
| BPaLC | 8 | 2 | 17 | 11.8 | 0 | 33 | 0.0 | 13 | 47 | 27.7 | 15 | 97 | 15.5 |
| BPaLC | 12 | 2 | 15 | 13.3 | 1 | 33 | 3.0 | 9 | 46 | 19.6 | 12 | 94 | 12.8 |
| BPaLC | 16 | 4 | 14 | 28.6 | 6 | 32 | 18.8 | 10 | 44 | 22.7 | 20 | 90 | 22.2 |
| BPaLC | 20 | 4 | 13 | 30.8 | 4 | 31 | 12.9 | 11 | 41 | 26.8 | 19 | 85 | 22.4 |
| BPaLC | 24 | 3 | 13 | 23.1 | 3 | 27 | 11.1 | 13 | 43 | 30.2 | 19 | 83 | 22.9 |

NOTE: BY: Belarus, SA: South Africa. UZ: Uzbekistan. W: week.

|  |  | BY | | | SA | | | UZ | | | OVERALL | | |
| --- | --- | --- | --- | --- | --- | --- | --- | --- | --- | --- | --- | --- | --- |
| Arm | Visit/W | QTcF  >450 | N | % | QTcF  >450 | N | % | QTcF  >450 | N | % | QTcF  >450 | N | % |
| BPaL | 0 | 0 | 20 | 0.0 | 0 | 35 | 0.0 | 0 | 52 | 0.0 | 0 | 107 | 0.0 |
| BPaL | 1 | 0 | 20 | 0.0 | 0 | 34 | 0.0 | 4 | 51 | 7.8 | 4 | 105 | 3.8 |
| BPaL | 2 | 0 | 20 | 0.0 | 1 | 32 | 3.1 | 6 | 52 | 11.5 | 7 | 104 | 6.7 |
| BPaL | 3 | 0 | 18 | 0.0 | 0 | 27 | 0.0 | 11 | 49 | 22.4 | 11 | 94 | 11.7 |
| BPaL | 4 | 2 | 19 | 10.5 | 0 | 32 | 0.0 | 7 | 51 | 13.7 | 9 | 102 | 8.8 |
| BPaL | 5 | 1 | 17 | 5.9 | 0 | 25 | 0.0 | 7 | 47 | 14.9 | 8 | 89 | 9.0 |
| BPaL | 6 | 1 | 17 | 5.9 | 0 | 24 | 0.0 | 10 | 46 | 21.7 | 11 | 87 | 12.6 |
| BPaL | 7 | 1 | 17 | 5.9 | 0 | 24 | 0.0 | 8 | 47 | 17.0 | 9 | 88 | 10.2 |
| BPaL | 8 | 0 | 18 | 0.0 | 0 | 30 | 0.0 | 9 | 49 | 18.4 | 9 | 97 | 9.3 |
| BPaL | 12 | 1 | 18 | 5.6 | 0 | 27 | 0.0 | 7 | 47 | 14.9 | 8 | 92 | 8.7 |
| BPaL | 16 | 2 | 17 | 11.8 | 1 | 29 | 3.4 | 2 | 40 | 5.0 | 5 | 86 | 5.8 |
| BPaL | 20 | 0 | 17 | 0.0 | 0 | 29 | 0.0 | 6 | 41 | 14.6 | 6 | 87 | 6.9 |
| BPaL | 24 | 3 | 17 | 17.6 | 1 | 27 | 3.7 | 3 | 40 | 7.5 | 7 | 84 | 8.3 |

NOTE: BY: Belarus, SA: South Africa. UZ: Uzbekistan. W: week

# A5 Additional plots

Figures S1, S2 and S3, show graphical displays of QTcF measured over the 108-week trial follow-up period, by investigational arms. Figure S4 shows the proportion of QTcF measures >450ms over the 24-week treatment period, by investigational arms.

**Figure S1: Spaghetti plots over 108 weeks**


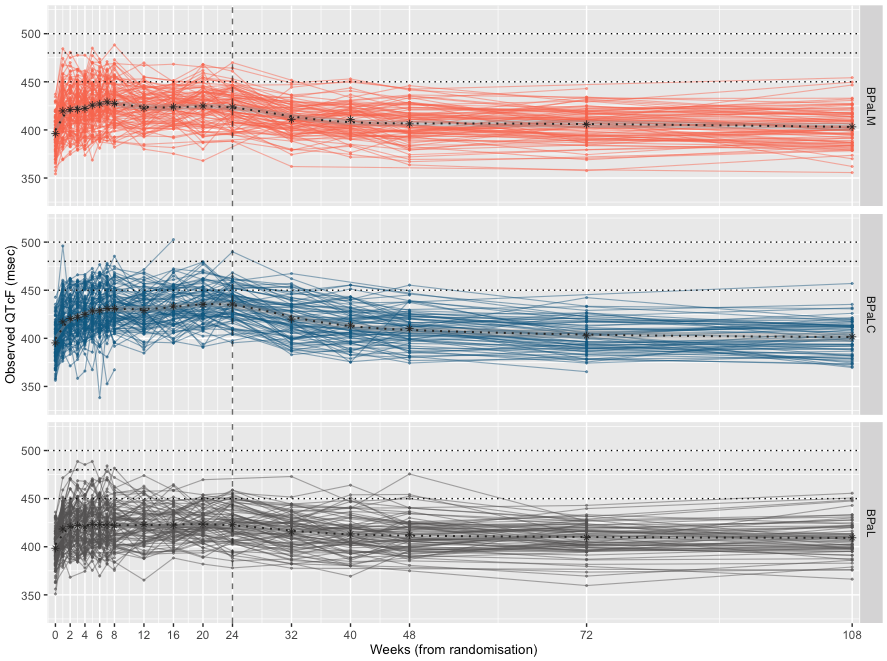


**Figure S2: Boxplots plots of observed QTcF over 108 weeks**


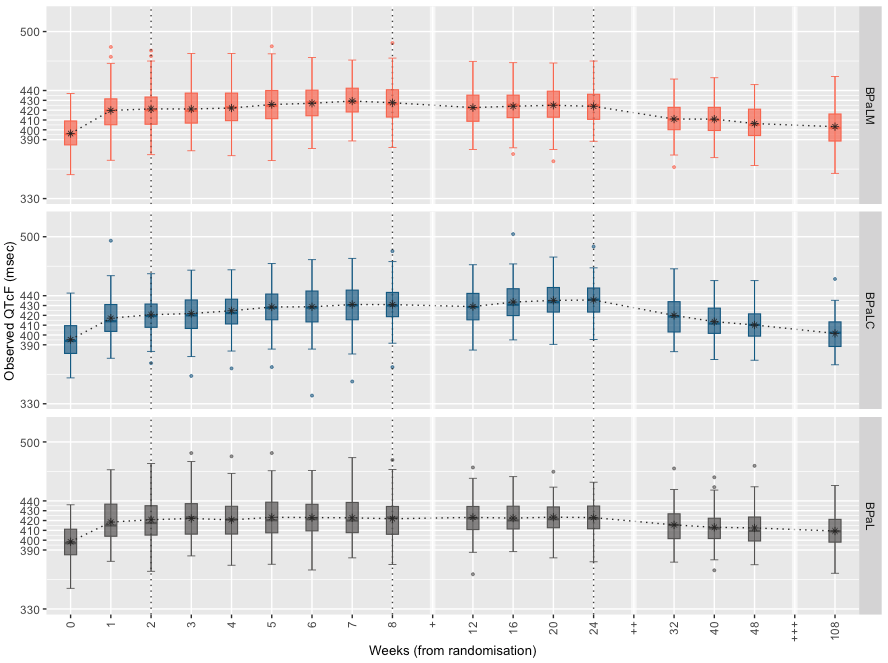


**Figure S3: Boxplot of QTcF differences from baseline per arm**


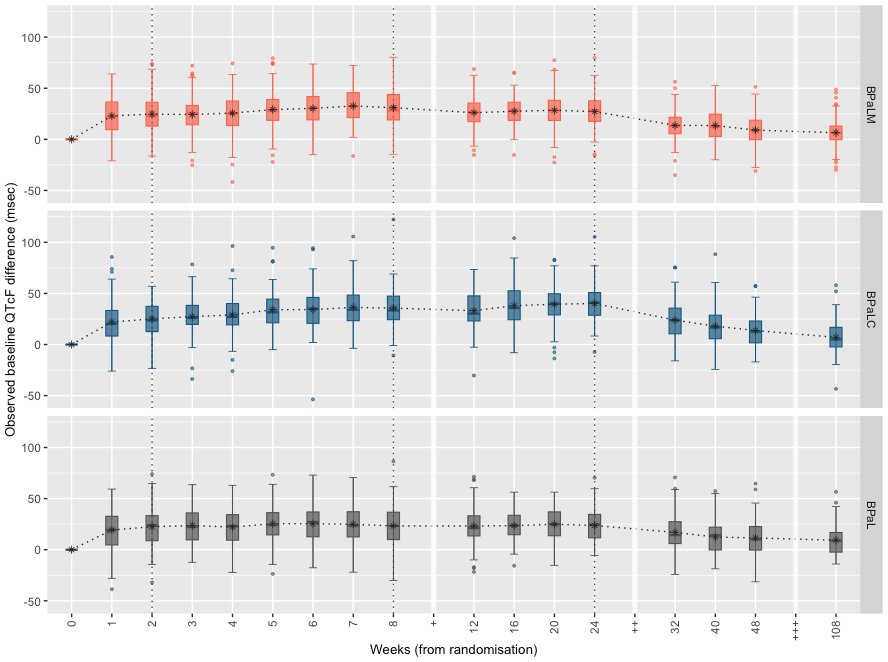


**Figure S4: proportion of QTcF measures >450ms by week and study arm**


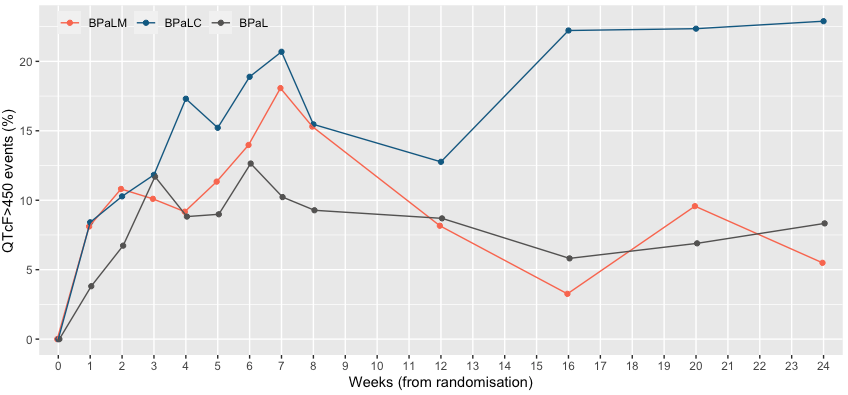


# A6 Longitudinal model for QTcF, output and diagnostics

Longitudinal model for continuous QTcF outcome: output (Table S1) and diagnostics (Figure S5).

**Table S1: Model output**

| Fixed effects |  |  |  |  |  |  |
| --- | --- | --- | --- | --- | --- | --- |
| Parameter | Coefficient | SE | CI_low | CI_high | t-statistic | p-value |
| (Intercept) | 422.52 | 2.28 | 418.04 | 427 | 185.08 | < .001 |
| visit wk n1 | -0.25 | 0.03 | -0.3 | -0.19 | -8.89 | < .001 |
| visit wk n2 | -0.57 | 0.88 | -2.3 | 1.16 | -0.65 | 0.515 |
| rand reg Regimen 2 (BPaLC) | 8.28 | 2.11 | 4.15 | 12.41 | 3.93 | < .001 |
| rand reg Regimen 1 (BPaLM) | 2.42 | 2.09 | -1.67 | 6.51 | 1.16 | 0.246 |
| country SA | -10.82 | 2.38 | -15.47 | -6.16 | -4.55 | < .001 |
| country UZ | 6.91 | 2.26 | 2.49 | 11.34 | 3.06 | 0.002 |
| visit wk n1 × BPaLC | 0.03 | 0.04 | -0.05 | 0.1 | 0.66 | 0.507 |
| visit wk n1 × BPaLM | -0.05 | 0.04 | -0.13 | 0.03 | -1.3 | 0.194 |
| BPaLC × visit wk n2 | 6.03 | 1.24 | 3.59 | 8.47 | 4.85 | < .001 |
| BPaLM × visit wk n2 | -0.30 | 1.23 | -2.71 | 2.11 | -0.24 | 0.809 |

Note: ‘visit wk n1’: 1/time^2^ and ‘visit wk n2: ln(time). BY: Belarus, SA: South Africa. UZ: Uzbekistan

| Random effects |  |
| --- | --- |
| Parameter | Coefficient |
| SD (Intercept: subjectid) | 14.97 |
| SD (visit.wk.n1: subjectid) | 0.24 |
| SD (visit.wk.n2: subjectid) | 7.34 |
| Cor (Intercept~visit.wk.n1: subjectid) | -0.16 |
| Cor (Intercept~visit.wk.n2: subjectid) | -0.04 |
| Cor (visit.wk.n1~visit.wk.n2: subjectid) | 0.91 |
| SD (Residual) | 10.15 |
| Note: ‘visit wk n1’: 1/time^2^ and ‘visit wk n1’: ln(time) | |

| **Figure S5: Diagnostics summary** |  |
| --- | --- |
| Normality of residuals | Caterpillar plot of random effects |
| 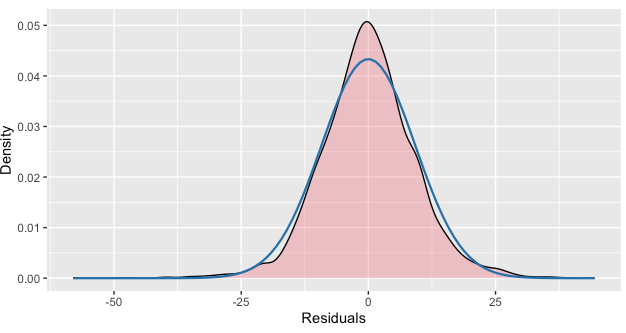 | 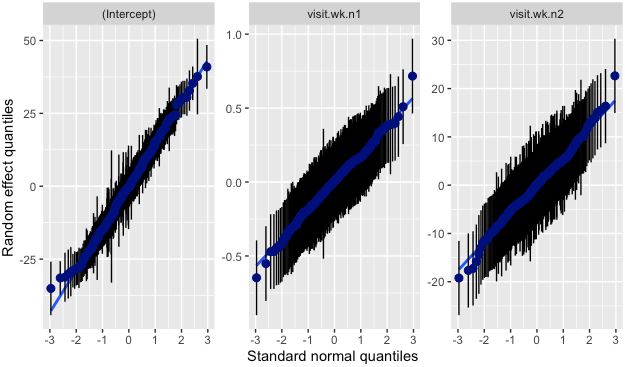 |
| Normality of residuals and ouliers | Homoscedasticy |
| 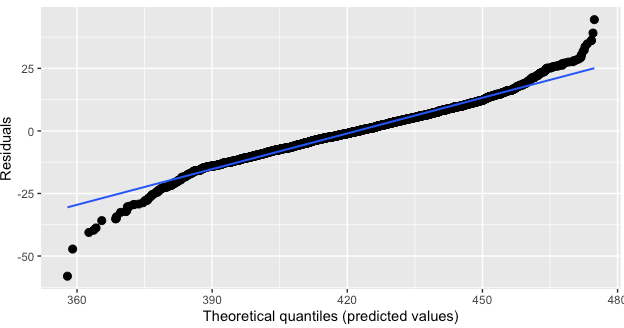 | 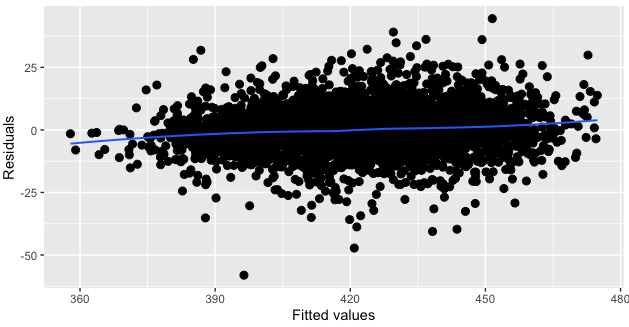 |

Plots show reasonably normally distributed random effects and relatively constant variance across the fitted range.

# A7 Standard of Care Details

Distribution of type of SOC across countries

| SOC type | BY N=19 | SA N=38 | UZ N=52 | Total N=109 | p.value |
| --- | --- | --- | --- | --- | --- |
| Long_new | 10 (52.6%) | 11 (28.9%) | 15 (28.8%) | 36 (33.0%) |  |
| Long_old | 9 (47.4%) | 1 (2.6%) | 23 (44.2%) | 33 (30.3%) |  |
| Short_new | 0 (0.0%) | 25 (65.8%) | 2 (3.8%) | 27 (24.8%) |  |
| Short_old | 0 (0.0%) | 1 (2.6%) | 12 (23.1%) | 13 (11.9%) |  |

NOTE: BY: Belarus, SA: South Africa. UZ: Uzbekistan.

Categorisation of SOC variable

|  | SOC not current | SOC current |
| --- | --- | --- |
| SOC long | 33 | 36 |
| SOC short | 13 | 27 |

# A8 Model results for objective 2 in SOC arm (country, age and BMI)


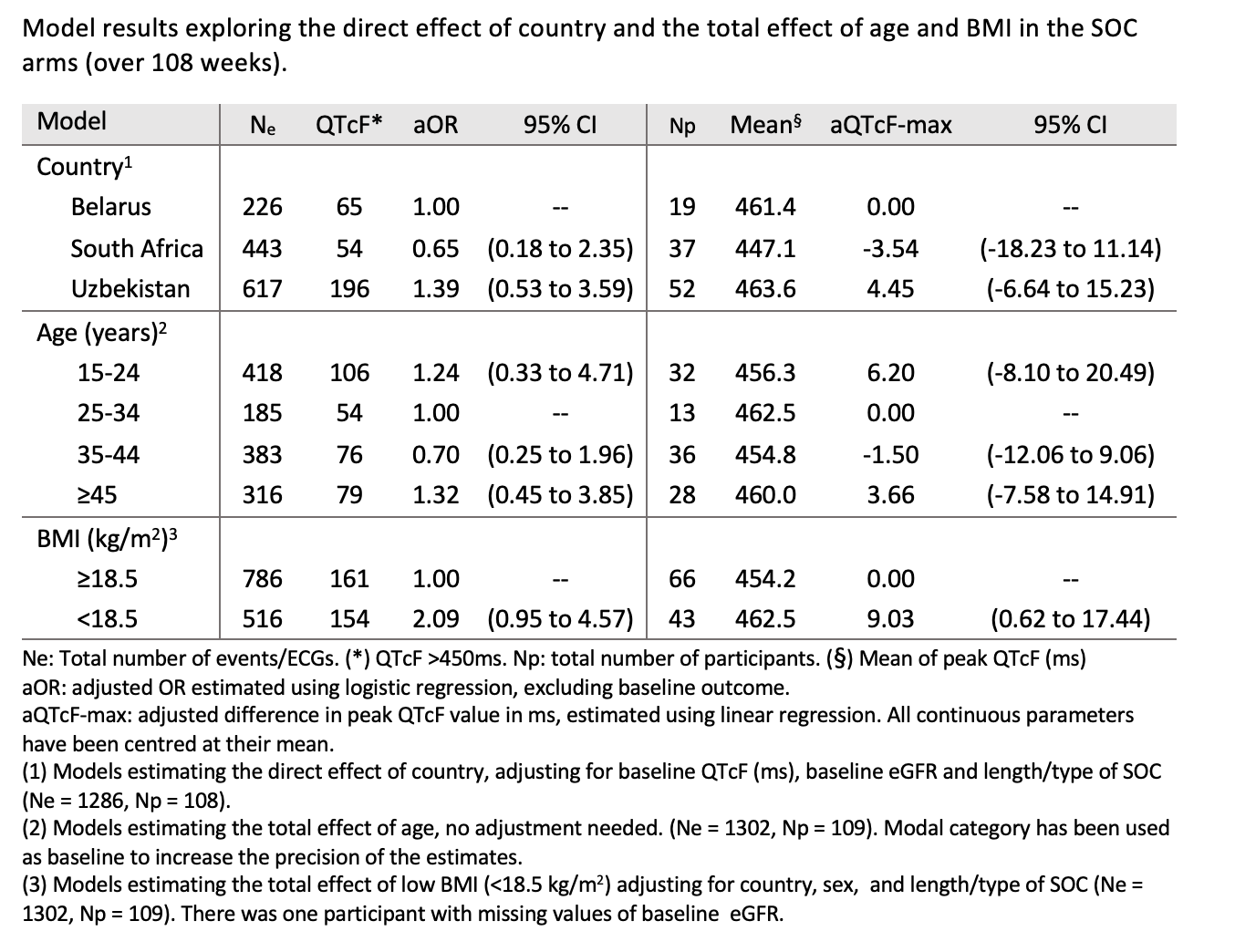

Supplement: Supplemental material — Appendices A1, A2, A4 to A8; Fig. S1 to S5. [file aac.00536-24-s0002.docx]
